# CXXC5 mitigates *P. gingivalis*-inhibited cementogenesis by influencing mitochondrial biogenesis

**DOI:** 10.1186/s12964-023-01283-1

**Published:** 2024-01-02

**Authors:** Li Ma, Huiyi Wang, Xin Huang, Hantao Huang, Yan Peng, Heyu Liu, Xiaoxuan Wang, Zhengguo Cao

**Affiliations:** 1 State Key Laboratory of Oral & Maxillofacial Reconstruction and Regeneration, Key Laboratory of Oral Biomedicine Ministry of Education, Hubei Key Laboratory of Stomatology, School & Hospital of Stomatology, Wuhan University, Wuhan, China; 2 Department of Periodontology, School & Hospital of Stomatology, Wuhan University, Wuhan, China

**Keywords:** *P. gingivalis*, CXXC5, Cementoblast, Cell differentiation, Mitochondrial biogenesis, PGC-1α

## Abstract

**Background:**

Cementoblasts on the tooth-root surface are responsible for cementum formation (cementogenesis) and sensitive to *Porphyromonas gingivalis* stimulation. We have previously proved transcription factor CXXC-type zinc finger protein 5 (CXXC5) participates in cementogenesis. Here, we aimed to elucidate the mechanism in which CXXC5 regulates *P. gingivalis-*inhibited cementogenesis from the perspective of mitochondrial biogenesis.

**Methods:**

In vivo, periapical lesions were induced in mouse mandibular first molars by pulp exposure, and *P. gingivalis* was applied into the root canals. In vitro, a cementoblast cell line (OCCM-30) was induced cementogenesis and submitted for RNA sequencing. These cells were co-cultured with *P. gingivalis* and examined for osteogenic ability and mitochondrial biogenesis. Cells with stable CXXC5 overexpression were constructed by lentivirus transduction, and PGC-1α (central inducer of mitochondrial biogenesis) was down-regulated by siRNA transfection.

**Results:**

Periapical lesions were enlarged, and PGC-1α expression was reduced by *P. gingivalis* treatment. Upon apical inflammation, *Cxxc5* expression decreased with* Il-6* upregulation. RNA sequencing showed enhanced expression of osteogenic markers, *Cxxc5*, and mitochondrial biogenesis markers during cementogenesis. *P. gingivalis* suppressed osteogenic capacities, mitochondrial biogenesis markers, mitochondrial (mt)DNA copy number, and cellular ATP content of cementoblasts, whereas CXXC5 overexpression rescued these effects. PGC-1α knockdown dramatically impaired cementoblast differentiation, confirming the role of mitochondrial biogenesis on cementogenesis.

**Conclusions:**

CXXC5 is a *P. gingivalis*-sensitive transcription factor that positively regulates cementogenesis by influencing PGC-1α-dependent mitochondrial biogenesis.

Video Abstract

**Supplementary Information:**

The online version contains supplementary material available at 10.1186/s12964-023-01283-1.

## Background

Periodontitis and apical periodontitis (AP) are common oral infectious diseases, which share the same pathogenic bacteria (*Porphyromonas gingivalis*) and similar features (e.g., periapical inflammation and cementum destruction) [1, 2]. During normal development, cementum matrix is deposited on the tooth-root surface by cementoblasts in a process known as “cementogenesis”. In diseased conditions, cementoblasts are exposed to inflammatory environments and interact with downward-invading pathogens, and their properties are adversely affected [3, 4]. Specifically, the expression of mineralization markers osteocalcin (OCN) and bone sialoprotein (BSP) in cementoblasts is altered by *P. gingivalis* lipopolysaccharide [5]. Therefore, more attention should be paid to cementoblast differentiation or cementogenesis under pathological bacterial stimulation.

Our group has long focused on the transcription factors associated with cementogenesis, such as Osterix and CXXC-type zinc finger protein 5 (CXXC5), and they are remarkedly decreased when cementoblasts receive *P. gingivalis* treatment in vitro [6–8]. Besides cementoblast differentiation, CXXC5 is also involved in the differentiation of osteoblasts, endothelial cells, and oligodendrocytes [9–11]. Recently, researchers have revealed active mitochondria support osteogenic differentiation of bone marrow stromal cells, and that mitochondrial dysfunction is triggered by *P. gingivalis* in endothelial cells [12, 13]. By now, the participation of subcellular-structure organelles in cementogenesis has been largely overlooked, although they may act as downstream effectors of the aforementioned transcription factors. Early in 1990, investigators demonstrated that active cementoblasts responsible to form new cementum matrix are abundant in intracellular organelles, including rough endoplasmic reticula, Golgi complexes, and in particular, mitochondria [14]. Therefore, how mitochondria participate in cementum formation and cementum destruction aroused our curiosity.

During the migration, proliferation, differentiation, and mineralization of cells, a constant supply of adenosine triphosphate (ATP) is urgently needed, and the powering sources of cells (mitochondria) are undoubtedly involved [12, 15]. By regulating the number and function of mitochondria, cells respond to different developmental signals or adapt to various environmental stress [16, 17]. Mitochondrial biogenesis (generation of new mitochondria) is provoked physiologically to compensate for the additional energy demand, while disturbed mitochondrial biogenesis is correlated with severe pathologic conditions, such as diabetes mellitus, neurodegeneration, and cardiovascular diseases [18–20]. In short, the biogenesis process is mainly mediated by peroxisome proliferator-activated receptor-γ coactivator 1 alpha (PGC-1α), nuclear respiratory factor 1 (NRF1), NRF2, and mitochondrial transcription factor A (TFAM) [21, 22]. The activation of the PGC-1α/NRF/TFAM axis results in the synthesis of mitochondrial (mt)DNA and proteins, and the generation of new mitochondria.

We aimed to elucidate the predominant role of mitochondrial biogenesis in cementogenesis and a novel mechanism on how CXXC5 mediates *P. gingivalis*-suppressed cementoblast differentiation. Our research innovatively verified that CXXC5 expression decreased under *P. gingivalis* stimulation, which affected mitochondrial biogenesis through the PGC-1α/NRF1/TFAM axis, thereby inhibiting cementoblast differentiation.

## Materials and methods

Materials and methods concerning quantitative real-time polymerase chain reaction (qPCR) and western blotting are presented in the Appendix. Information on reagent sources and identifiers is shown in Appendix Table 1.

### Bacterial preparation

*P. gingivalis* strain ATCC 33277 was maintained in the Microbiology Laboratory, School of Stomatology, Wuhan University. Bacteria were cultured in blood agar plates or trypticase soy broth (TSB) in an anaerobic tank (80% N_2_, 10% H_2_, and 10% CO_2_). Yeast extracts (0.1%), menadione (1 μg/mL), and hemin (5 μg/mL) were added to TSB, where the single colony of *P. gingivalis* proliferated to the log phage. Bacterial counts were determined by spectrophotometry (OD_600nm_ 1 = 10^9^ cells), and cell deposits were gathered by centrifugation at 6000 × *g* for 10 min at room temperature.

### Periapical lesion induction

6-week-old male C57BL/6 mice were randomly allocated into three groups (*n* = 12/group): healthy, AP, and AP plus *P. gingivalis* group. Mice were anesthetized by sodium pentobarbital (1.3%, 0.1 mL/20 g), and the mandibular first molars were clearly exposed. Cavity preparation on the left molars was achieved with a high-speed dental handpiece, a no. 1/4 fissure bur, and a magnifying glass. Half of the mice were further treated with repeat oral application of *P. gingivalis* (MWF/week) in 2% carboxymethylcellulose vehicle. Pulp chambers were open for 14 or 21 days before mouse execution and mandible sampling. The right mandibular samples of *P. gingivalis*-free mice served as healthy controls. The mandibles were separated for histological and radiographic analysis. Specifically, five pairs of bilateral periapical tissues were sampled for qPCR to measure the expression of interleukin-6 (*Il-6*) and *Cxxc5*.

### Immunofluorescence

Mandible specimens were standardly fixed with 4% paraformaldehyde, decalcified by EDTA, and embedded in paraffin. 5- μm- thick slices were successively incubated with goat serum at 37℃ for 1 h, primary antibodies (anti-*P. gingivalis*, 5 μg/mL; DSHB or anti-PGC-1α, 1:200 dilution; NOVUS) overnight at 4℃, secondary antibodies conjugated to fluorescein isothiocyanate (1:200; Servicebio) or Cy3 (1:200; Servicebio) at 37℃ for 1 h and, ultimately, mounted with 4′,6-diamidino-2-phenylindole for 10 min. The expression of each indicator in apical areas was photographed using a fluorescence microscope (Olympus IX83).

### Radiography and micro-computed tomography (micro-CT)

After mandibles had been dissected, X-ray photography was undertaken using the In-Vivo DXS PRO system(Bruker) to rule out samples with insufficient unfolding of the pulp chamber roof or perforation of the pulp chamber floor, and to preliminarily assess the ranges of periapical-bone destruction. To capture clearer images of apical lesions, these mandibles were scanned by Quantum GX micro-CT (PerkinElmer), and images were captured in SimpleViewer. The parameters were set as 70 kV, 114 μA, with a resolution of 20 μm.

### Cell culture and RNA sequencing

In vitro, a murine cementoblast cell line (mycoplasma-free OCCM-30) was cultured in high-glucose Dulbecco’s modified Eagle’s medium containing 10% fetal bovine serum (FBS) at 37℃ in a humidified atmosphere of 5% CO_2_. Osteogenic induction medium (OIM) containing 5% FBS, ascorbic acid (50 μg/mL), and Naβ-glycerophosphate (10 mM) was also employed.

OCCM-30 cells were cultured in OIM for 0 or 7 days. The total RNA of these cells was extracted and submitted for sequencing in Annoroad. Reads per kilobase per million mapped reads was used as an indicator for differences in gene expression. Typical osteogenic genes, mitochondrial biogenesis markers, and *Cxxc5* were selected for heatmap creation.

### Lentivirus transduction and RNA interference (RNAi)

To overexpress *Cxxc5*, OCCM-30 cells with > 50% confluence were infected by normal control (NC)-over or Cxxc5-over lentivirus (Hanbio) with the assistance of polybrene for 8 h. Cells were incubated continuously with puromycin and examined under a fluorescence microscope to ensure adequate infection efficiency.

For knockdown of the gene for PGC-1α (*Ppargc1a*), cells were cultured to 70% confluence. Two sequences from GenePharma were tested: si1 (5'-AAG ACG GAU UGC CCU CAU UUG TT-3'/5'-CAA AUG AGG GCA AUC CGU CUU TT-3'), and si2 (5'-GUA GCG ACC AAU CGG AAA UTT-3'/5'-AUU UCC GAU UGG UCG CUA CTT-3'). The INTERFERin transfection reagent was used following manufacturer’s instructions.

### Cell proliferation assay

The effect of stable *Cxxc5* overexpression on cementoblast proliferation was evaluated by Cell Counting Kit-8 (CCK-8) assays at an initial seeding density of 1000 cells/well in 96-well plates. Fresh medium containing 10% CCK-8 reagent was added to microplates at fixed times for 1 week, and the mixture was incubated for 1.5 h before final measurement of absorbance at 450 nm.

### Alkaline phosphatase (ALP) staining and Alizarin red staining (ARS)

Cells were seeded in six-well plates, induced osteogenic differentiation for 7 and 14 days, washed with phosphate-buffered saline, and prefixed by 4% paraformaldehyde for 15 min. ALP staining solution was prepared and applied according to protocol of the BCIP/NBT ALP Color Development Kit (Beyotime). To monitor mineral nodules, cells were stained with 1% alizarin red solution (pH 4.2) at 37 °C and photographed. Then, the reddish-brown nodules were dissolved thoroughly in 10% cetylpyridinium chloride and quantified at 562 nm by a microplate reader. Images were preserved with a camera and a microscope.

### Cellular ATP activity

To quantify cellular ATP levels, cells that received different treatments were digested and reseeded in 96-well black plates (20,000–30,000 cells/well). Cells were allowed to stabilize for 4 h, and the CellTiter-Glo® Luminescent Cell Viability Assay Kit (Promega) was used to induce cell lysis and release cellular ATP. Luminescence signals were recorded by a multimode microplate reader (Tecan) and normalized to cell numbers.

### Statistical analysis

Experimental data were representative of three separate experiments, analyzed with SPSS 16.0 and GraphPad Prism 8.0, and expressed as mean ± SD. Paired *t*-test, unpaired *t*-test, or one-way analysis of variance was used to evaluate the statistical differences, which were marked with “*” for *P* < 0.05 or “**” for *P* < 0.01. *P* > 0.05 was not labeled in the figures.

## Results

### *P. gingivalis* aggravated AP in mouse models, together with inhibition of CXXC5 and PGC-1α expression

**Fig. 1:**
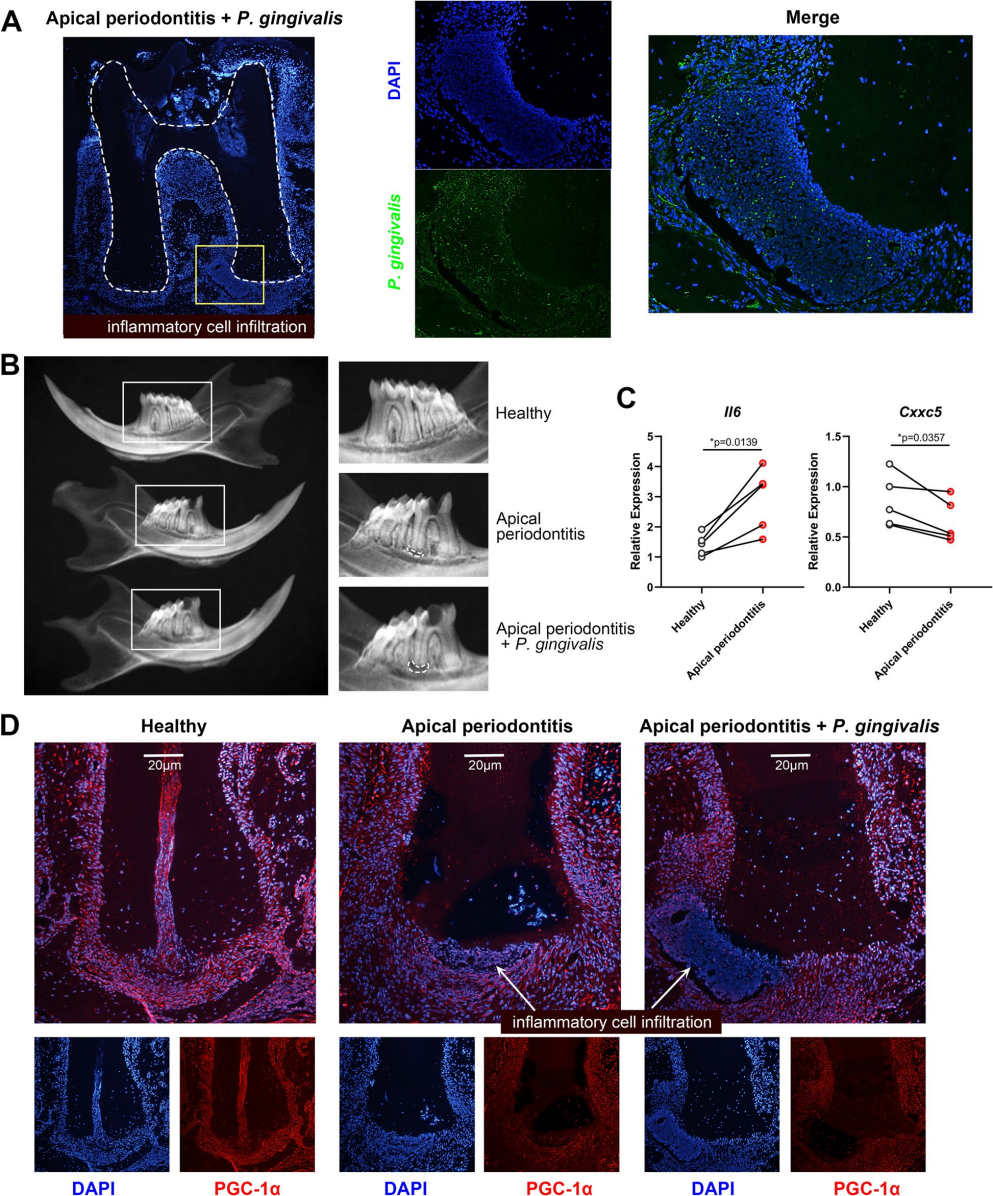
CXXC5 expression and mitochondrial biogenesis are suppressed in *P. gingivalis-*exacerbated apical lesions. **A** Immunofluorescence images showed unfolding of pulp chamber and evident inflammatory infiltration near the root cementum. *P. gingivalis* was orally applied and captured with green signals (*n* = 3). **B** Mandibles from healthy, AP, and AP plus *P. gingivalis* groups underwent radiography (*n* = 5). **C** Apical tissues were extracted from five mice (left pulp-exposed teeth compared with right intact teeth), and expression of *Il-6* and *Cxxc5* was measured by qPCR (*n* = 5). Individual values and *P* values of the paired *t*-test are present. **D** PGC-1α expression in apical areas was evaluated by immunofluorescence (*n* = 3). White arrows indicate inflammatory cell infiltration

In vivo, AP models were successfully established by pulp exposure. Immunofluorescence revealed that oral application of *P. gingivalis* suspension enabled bacterial invasion into apical areas (Fig. 1A). In contrast to the healthy group, the AP group exhibited significant apical bone destruction, and additional *P. gingivalis* treatment enlarged the ranges of bone resorption, as revealed by radiography (Fig. 1B and Appendix Figure 1A). Gene expression in periapical tissues between the right untreated molars with the left pulp-exposed molars from the same mouse was compared, and we discovered that expression of proinflammatory chemokine *Il-6* was upregulated, whereas that of transcription factor *Cxxc5* was downregulated (Fig. 1C). Considering that PGC-1α is a central indicator of mitochondrial biogenesis, its expression in apical cementoblasts and ambient periodontal ligament cells was evaluated. Fluorescence microscopy detected gradually quenched PGC-1α signals in the healthy, AP, and AP plus *P. gingivalis* groups (Fig. 1D).

Collectively, our results suggested that the expression of CXXC5 and PGC-1α was negatively correlated with apical inflammation and mineralized matrix destruction.

### Transcription factor CXXC5 mitigates *P. gingivalis*-inhibited cementogenesis

**Fig. 2:**
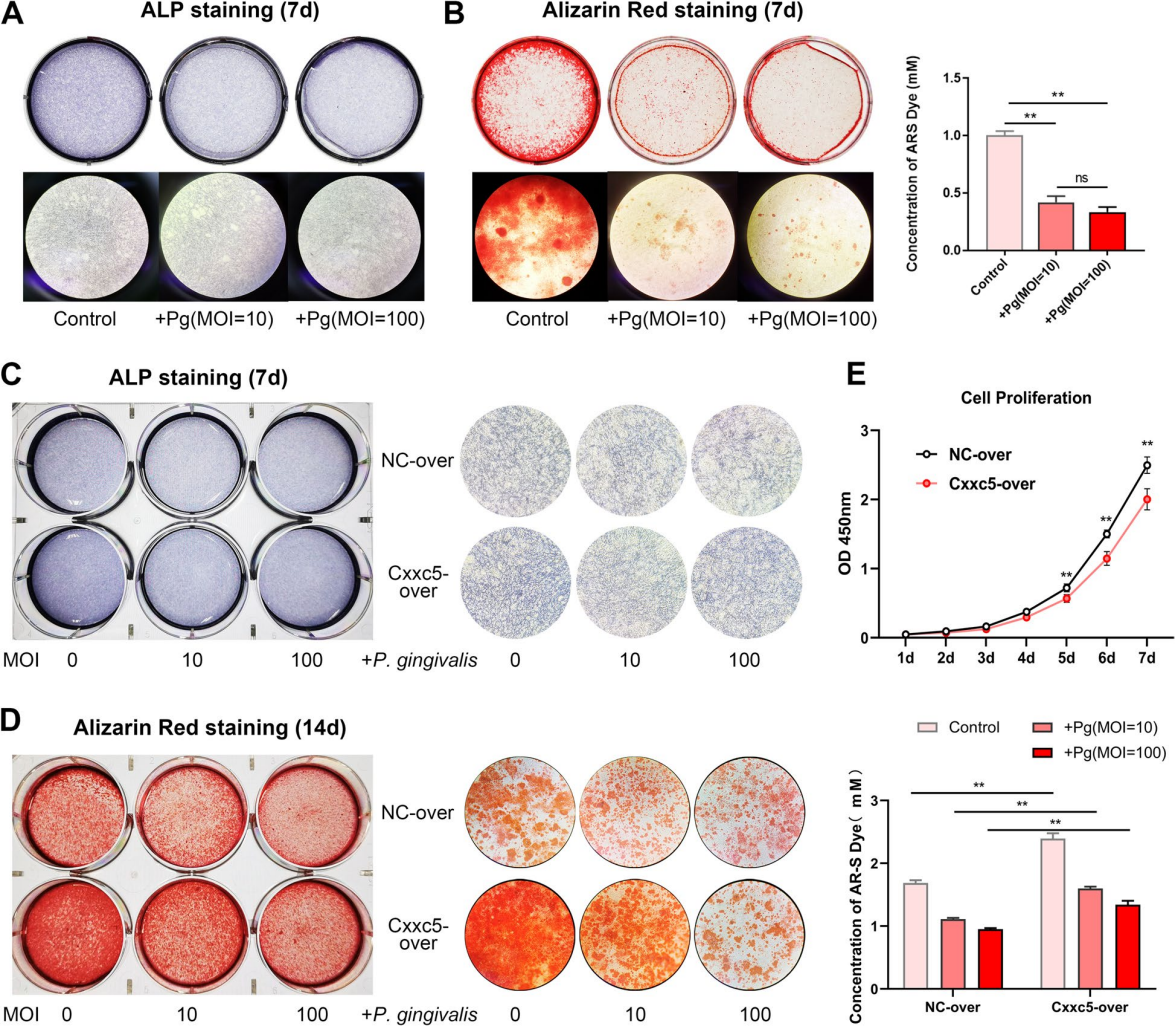
*P. gingivalis* significantly restrains cementoblast differentiation, and stable CXXC5 overexpression mitigates these osteogenic inhibitory effects. **A**, **B** Cementoblasts were co-cultured with *P. gingivalis* (multiplicity of infection [MOI] = 0, 10, 100) for 7 days, and ALP staining and ARS were performed. After image capture, mineralized nodules were dissolved in cetylpyridinium chloride and quantified at 562 nm (*n* = 3). **C**, **D** The co-culture system contained control or CXXC5-overexpressed cells and *P. gingivalis* (MOI = 0, 10, 100), and maintained for 7 and 14 days, with ALP staining and ARS (description and quantitation) done at selected time points (*n* = 3). **E** Cell proliferation of control and Cxxc5-over cementoblasts was monitored in normal culture medium for 7 days by CCK-8 assay (*n* = 5). Data are the mean ± SD. *P < 0.05, and **P < 0.01

In vitro, a cementoblast–*P. gingivalis* (multiplicity of infection [MOI] = 0, 10, 100) co-culture system was set up. On days 4 and 7, *P. gingivalis* elevated *Il-6* expression and declined OCN, Osterix, and BSP expression (Appendix Figure 2A and B). *P. gingivalis-*suppressed osteogenic capacities were observed more visually by ALP staining and ARS (Fig. 2A and B). Since the last part suggested a correlation between CXXC5 reduction and apical bone destruction, stable CXXC5-overexpression cell lines were obtained by lentivirus transduction (Appendix Figure 3A). Upon CXXC5 overexpression, ALP expression and mineralized nodules remarkedly increased, which largely rescued the inhibitory effects of *P. gingivalis* (Fig. 2C and D). CXXC5 also restrained cell proliferation slightly when evaluated in both normal medium and OIM (Fig. 2E and Appendix Figure 3B).

### Mitochondrial biogenesis is closely related to P. gingivalis-inhibited cementogenesis

**Fig. 3:**
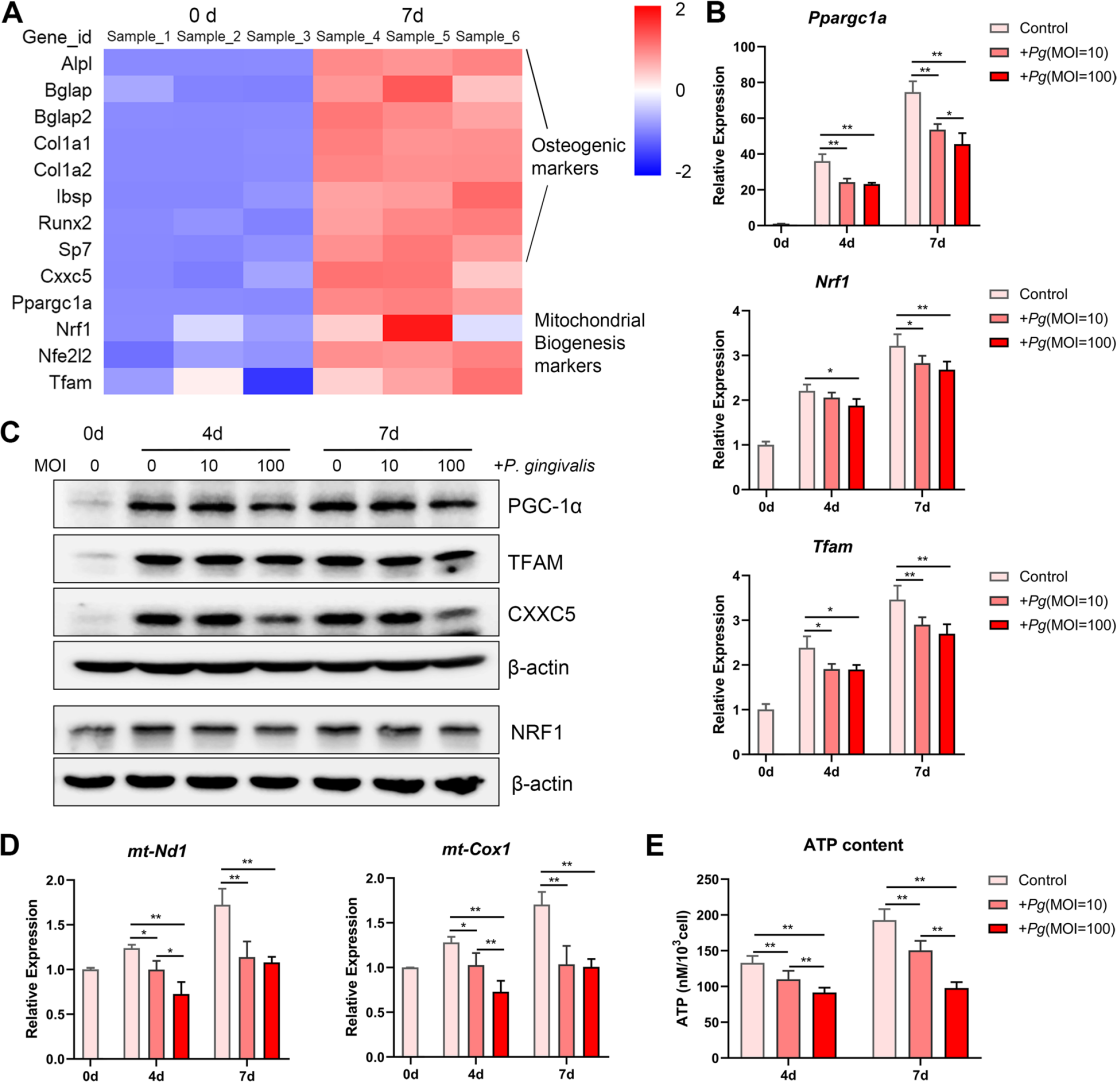
Mitochondrial biogenesis is closely related to cementoblast differentiation and *P. gingivalis* stimulation. **A** Similar expression trend was detected among osteogenic markers, *Cxxc5*, and mitochondrial biogenesis markers on days 0 and 7 of osteogenic induction, according to heatmaps (*n* = 3). **B**, **C** mRNA and protein levels of PGC-1α, NRF1, and TFAM were examined in the *P. gingivalis* co-culture system at designated time points by qPCR and western blotting (*n* = 3). **D** Expression of *mt-Nd1* and *mt-Cox1* was representative of mtDNA copy number (*n* = 3). **E** Cellular ATP was released by CellTiter-Glo reagents and quantified in a multimode microplate reader (*n* = 5). Data are the mean ± SD. *P < 0.05, and **P < 0.01

To explore the underlying mechanism of cementoblast differentiation, samples with osteogenic induction or not were prepared for RNA sequencing. Heatmaps showed that the expression of crucial markers of mitochondrial biogenesis (*Ppargc1a*, *Nrf1*, *Nfe2l2*, and *Tfam)* increased along with typical osteogenic markers and *Cxxc5* (Fig. 3A). These results were reinforced by qPCR and western blotting: the mRNA and protein levels of PGC-1α, NRF1, and TFAM increased with osteogenic induction but declined upon *P. gingivalis* stimulation (Fig. 3B and C)*.* Next, *mt-Nd1* and *mt-Cox1* were selected to represent mtDNA copy number, and cellular ATP content was detected to reveal mitochondrial functions. As expected, they were positively related to cementoblast differentiation and negatively related to bacterial treatment (Fig. 3D and E).

### CXXC5 works as an upstream regulator of PGC-1α-dependent mitochondrial biogenesis

**Fig. 4:**
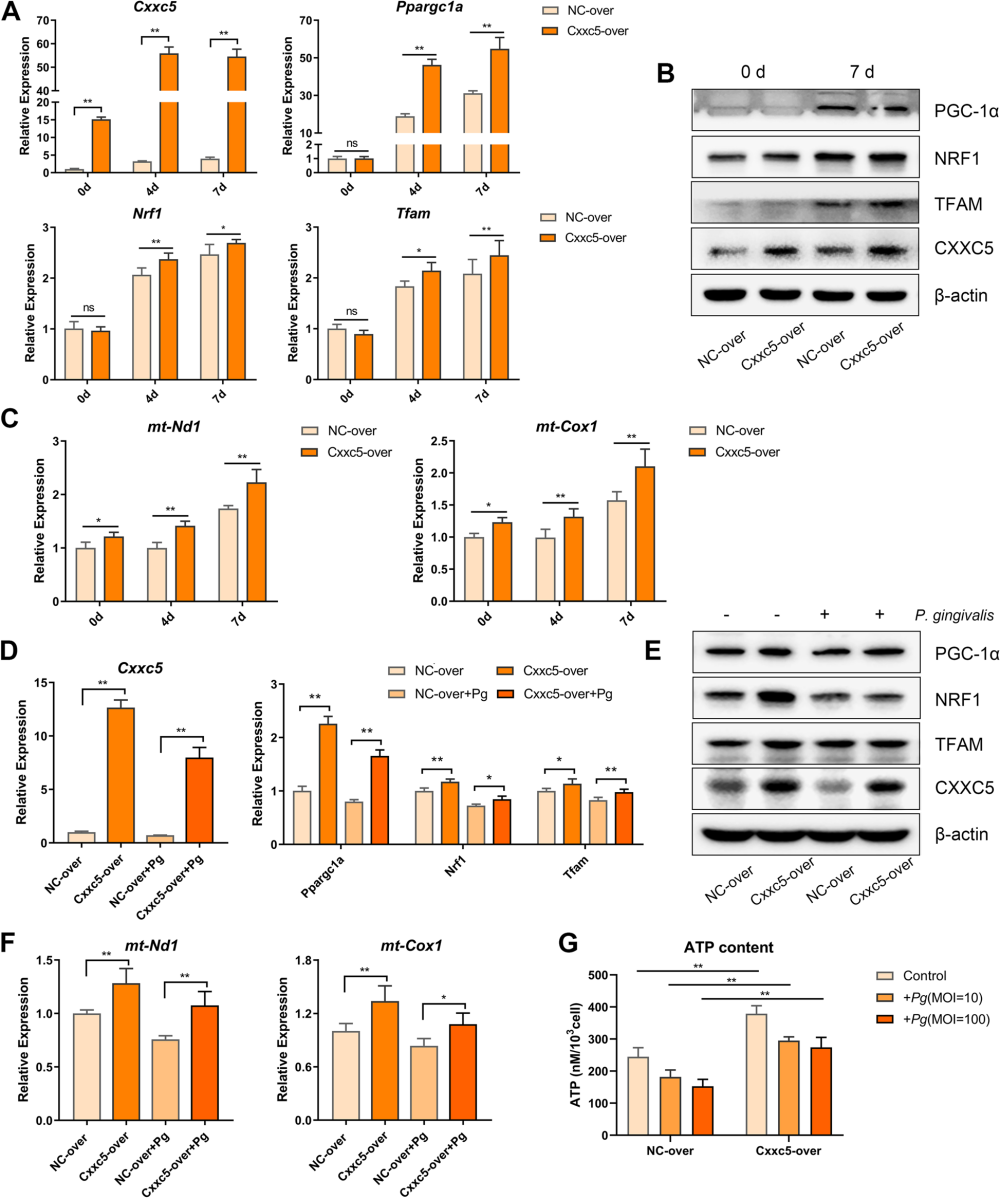
CXXC5 is an upstream modulator of PGC-1α-dependent mitochondrial biogenesis. **A**, **B** Expression of PGC-1α, NRF1, and TFAM was detected in NC-over and Cxxc5-over cells during osteogenic induction, by qPCR and western blotting (*n* = 3). **C** Expression of *mt-Nd1* and *mt-Cox1* was examined to represent changes in mtDNA copy number (*n* = 3). **D**, **E** Stable CXXC5-overexpressed cells were treated by *P. gingivalis* for 7 days, and rescued expression of PGC-1α, NRF1, and TFAM was testified by qPCR and western blotting (*n* = 3). **F**, **G** The mtDNA copy number and cellular ATP content were repressed by bacterial stimulation, and these effects were attenuated by CXXC5 overexpression (*n* = 3). Data are the mean ± SD. *P < 0.05, and **P < 0.01

Considering the collaborative rise of CXXC5 and mitochondrial biogenesis markers during cementoblast differentiation, and the simultaneous decrease of them upon bacterial irritation, their relationship was explored in NC-overexpressed (“NC-over”) and Cxxc5-overexpressed (“Cxxc5-over”) cementoblasts. First, when examined at different times and compared with the NC-over group, the expression of PGC-1α, NRF1, TFAM, *mt-Nd1*, and *mt-Cox1* increased gradually in the Cxxc5-over group (Fig. 4A–C). Next, in *P. gingivalis*-treated conditions, CXXC5 overexpression rescued the levels of *P. gingivalis*-inhibited mitochondrial biogenesis markers, mtDNA copy number, and cellular ATP content (Fig. 4D–F). Taken together, these data suggested CXXC5 was a vital and novel upstream mediator of PGC-1α-dependent mitochondrial biogenesis.

### CXXC5 mitigates *P. gingivalis*-inhibited cementogenesis by influencing mitochondrial biogenesis

**Fig. 5:**
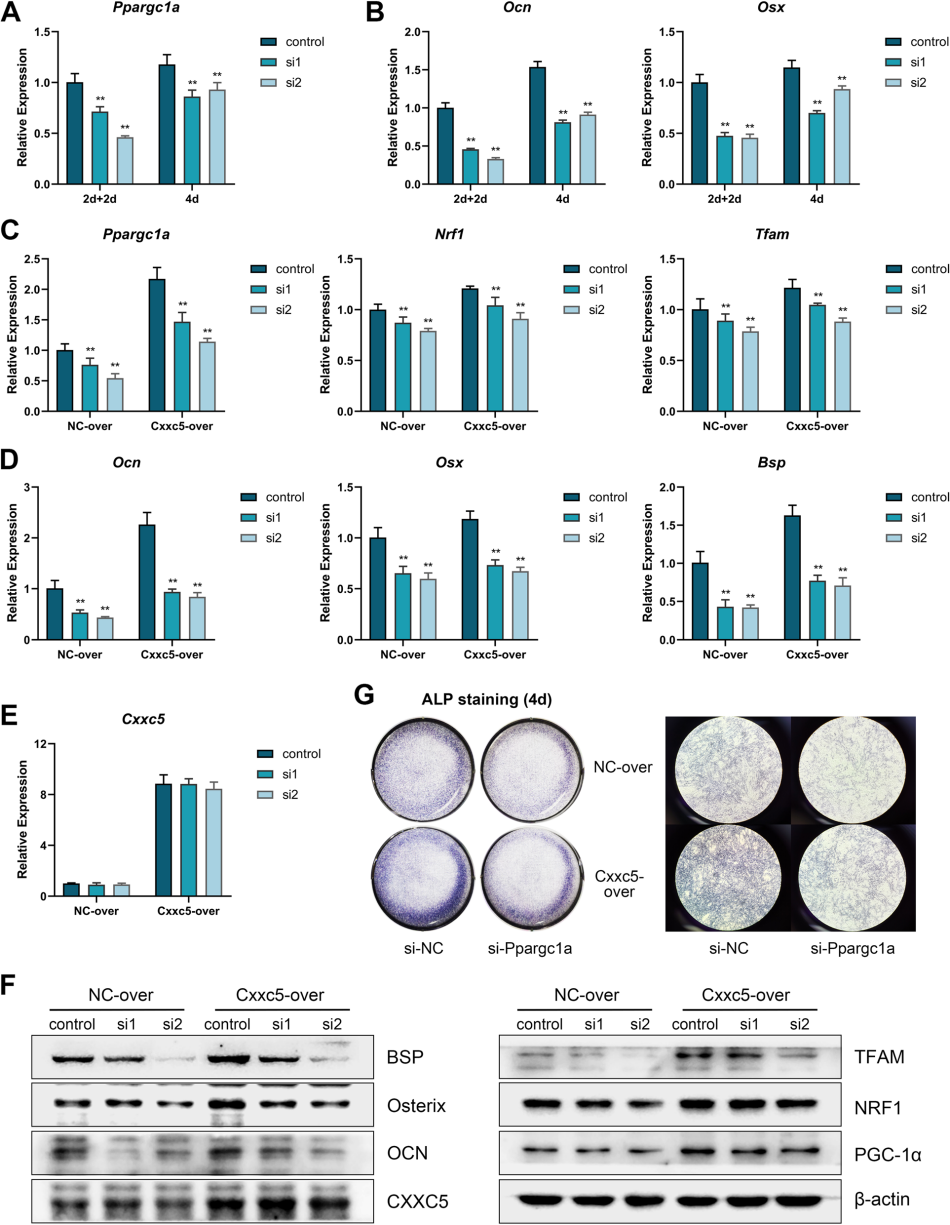
CXXC5 regulates cementogenesis by influencing mitochondrial biogenesis. **A**, **B** Two siRNAs for *Ppargc1a* were transfected transiently to OCCM cells for 4 days. The 2d + 2d group underwent secondary transfection on day 2. The suppressive effects of si-Ppargc1a on expression of *Ppargc1a*, *Ocn*, and *Osx* were revealed by qPCR (*n* = 3). **C**–**F** siRNAs were transfected to NC-over and Cxxc5-over cells in the “2d + 2d” method, and levels of osteogenic markers decreased with that of mitochondrial biogenesis markers, whereas CXXC5 expression was unchanged, shown by qPCR and western blotting (*n* = 3). **G** ALP staining was carried out on day 4 of siRNA transfection (*n* = 3). Data are the mean ± SD. *P < 0.05, and **P < 0.01

To validate the positive involvement of mitochondrial biogenesis in cementogenesis, the expression of PGC-1α was blocked by RNAi. Two siRNA sequences were transfected once (4d group) or twice (2d + 2d group) in 4 days to achieve higher knockdown efficiency. The twice-transfection method had more striking inhibitory effects on the expression of targeted *Ppargc1a*, subsequent *Nrf1/Tfam*, and downstream *Ocn*/*Osx/Bsp*, and was therefore employed in subsequent experiments (Fig. 5A and B and Appendix Figure 4A). These two pairs of siRNA also worked well in NC-over and Cxxc5-over cells, presented as stable and evident knockdown of the *Ppargc1a*/*Nrf1*/*Tfam* axis (Fig. 5C). As expected, the expression of osteogenic markers was dramatically repressed, whereas CXXC5 expression was relatively unaffected by RNAi (Fig. 5D–F). The deepened ALP staining in the Cxxc5-over group was also dodged upon si-Ppargc1a treatment (Fig. 5G)*.*

To summarize, mitochondrial biogenesis was an important link between the transcription factor CXXC5 and cementoblast differentiation. We uncovered a novel mechanism by which CXXC5 mitigates *P. gingivalis*-inhibited cementogenesis.

## Discussion

**Fig. 6:**
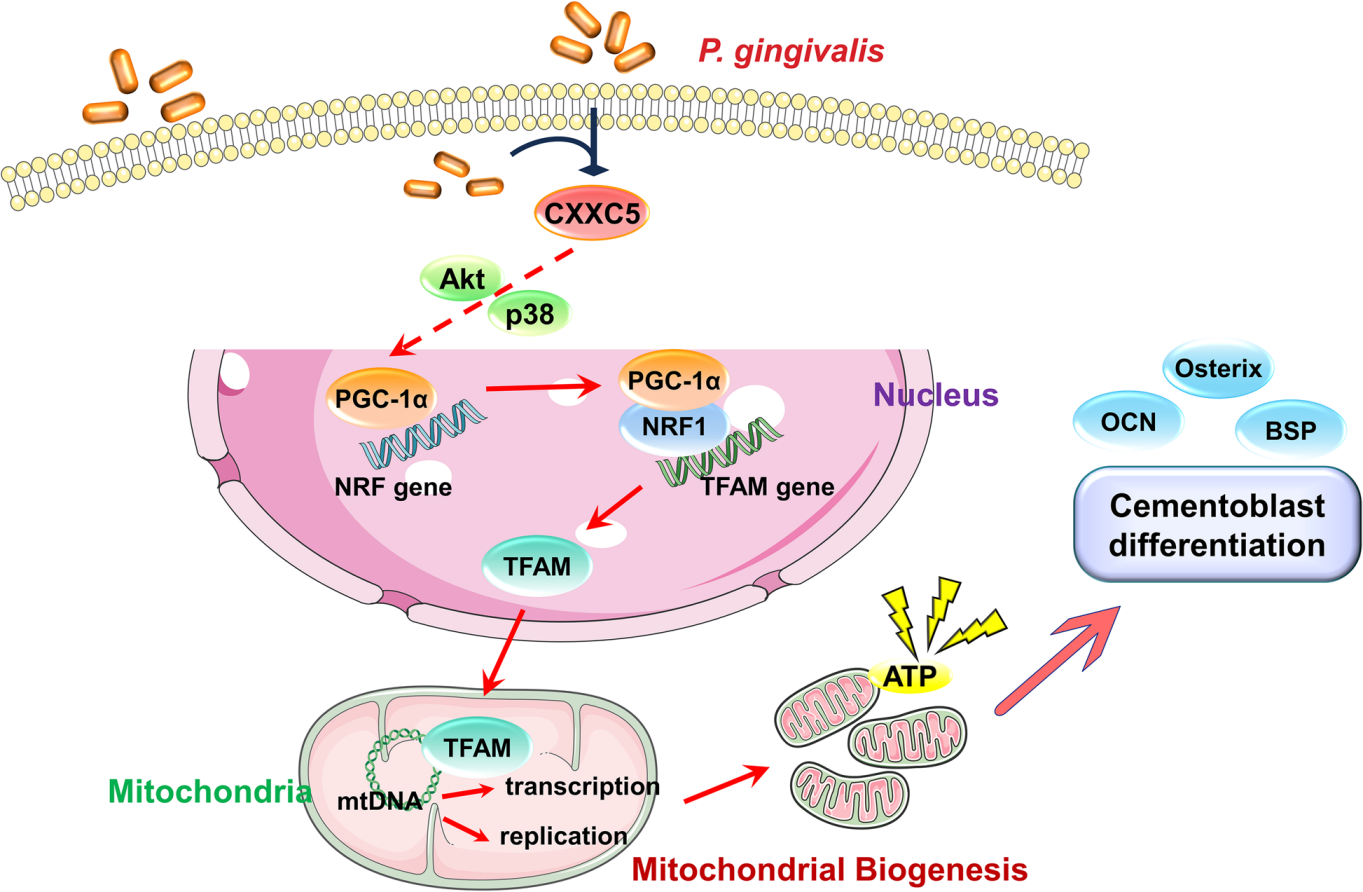
Graphical Abstract. CXXC5 expression decreased under *Porphyromonas gingivalis* stimulation, which negatively affected mitochondrial biogenesis through the PGC-1α/NRF1/TFAM axis, thereby inhibiting cementoblast differentiation

In this study, periodontal pathogen *P. gingivalis* decreased the expression of CXXC5 and PGC-1α and aggravated the range of apical lesions in mouse AP models. In vitro, CXXC5 overexpression rescued *P. gingivalis*-inhibited cementoblast differentiation, at least in part, by activation of the PGC-1α/NRF1/TFAM axis and mitochondrial biogenesis (Fig. 6).

Methodologically, gene overexpression is a well-established approach to explore gene functions, investigate biological pathways, and model diseases [23]. In previous studies, researchers have explored the functions of CXXC5 in various cell types by transient transfection, where the Cxxc5-over plasmids were constructed based on different vectors [7, 24, 25]. Compared with transient transfection generally used for short-term expression of gene products, stable transfection is advantageous and suitable for long-term pharmacological studies on genetic regulatory mechanisms, which is in line with the current study goals for cementoblast differentiation [26]. Therefore, the stable CXXC5-overexpression cell lines were developed by lentivirus transduction.

Impaired cementum formation is often associated with periodontal disease, chronic periapical inflammation, and conditions that compress the periodontal ligament, such as orthodontic pressure, tumor pressure, trauma, and impacted tooth [27]. Among them, periodontitis and AP are representative of inflammatory cementum resorption, which have higher prevalence than pressure-related ones and therefore become the focus of our research. Nevertheless, to investigate the interaction between *P. gingivalis* and cementoblasts in vivo, we chose a model of periapical lesions other than periodontitis, although *P. gingivalis* is the keystone pathogen of periodontal diseases [1].

In most rodent periodontitis models, scholars adopted a combination of *P. gingivalis* with ligatures, where deep periodontal pockets were formed, together with thicker gingiva and significant epithelial downgrowth [28–30]. One study involving only oral gavage of *P. gingivalis* detected *P. gingivalis* signals in gingival epithelial cells, periodontal ligament fibroblasts, and osteoblasts of the alveolar crest areas, but not cementoblasts [31]. These results suggested that the epithelial barrier against periodontal pathogens might still work in periodontitis models, which impeded direct *P. gingivalis*–cementoblasts contact in the lower one-third of tooth roots. In contrast, scholars successfully delivered *P. gingivalis* to periapical areas through the opened pulp chambers and canals of tooth [32]. Those findings encouraged us to establish periapical lesions with oral application of *P. gingivalis*. Despite we recognized *P. gingivalis* only is insufficient to represent various pathogens of periapical inflammation, and that a mixed-infection has superiority to reproduce the pathological processes, we are lacking in references about the composition and proportion of different pathogens in the mixed-infection model.

Bone and dental hard tissues (enamel, dentin, and cementum) are important mineralized structures in humans, and the mechanism of biomineralization is a current research “hotspot”. Recently, researchers have verified that mitochondria are involved in osteoblast differentiation or odontogenic differentiation, by means of mitochondrial biogenesis, mitochondrial dynamics, and mitophagy [33, 34]. Moreover, through artificial mitochondria transfer to bone marrow-derived mesenchymal stem cells, these cells successfully achieved enhanced osteogenesis in vitro and in cranial-defect models [15]. In contrast, no further exploration of mitochondria in cementoblasts has been carried out since abundant mitochondria were detected in active cementoblasts of newly-formed cementum matrix in 1990 [14]. Therefore, we attempted to elucidate the possible involvement of mitochondrial biogenesis in cementoblast differentiation to counter this knowledge gap.

Mitochondrial biogenesis is a process to provide energy, coordinated by both nuclear and mitochondrial genomes [17]. Based on our results, new mitochondria generation is vital for cementoblast differentiation, whereas the ATP-scavenging effect of intracellular *P. gingivalis* may occur during disease. The typical axis that regulates the biosynthesis of mitochondria is the PGC-1α/NRF/TFAM signaling cascade. Specifically, PGC-1α is the “master regulator”, while NRF1, NRF2, and TFAM regulate the synthesis of main mitochondrial enzymes and mtDNA [21, 35]. Considering NRF1 binding is the major determinant of *Tfam* promoter functions, our study emphasized NRF1 but not NRF2 [35]. Moreover, several studies indicated bidirectional regulation or feedback loop between PGC-1α and NRF2 [36]. Besides direct involvement in mitochondrial biogenesis, NRF2 also works as a powerful anti-inflammatory molecule and a defense mechanism to counteract reactive oxygen species (ROS) generation, while we have confirmed that the accumulation of ROS and inflammatory cytokines can be triggered by *P. gingivalis* in cementoblasts [8, 37, 38]. The observations stated above complicate the role of NRF2 in the *P. gingivalis*–cementoblast co-culture system, thus requiring further in-depth exploration.

The implication of transcription factor CXXC5 in cementogenesis and autophagy has been preliminarily explored by our research team previously, whereby common signaling pathways like MAPK, Akt, Stat3, and Wnt served as downstream mediators of CXXC5 [7, 8]. In the present study, CXXC5 was an upstream regulator of PGC-1α possibly through an indirect effect. Since CXXC5 positively regulated p38 MAPK and Akt activation, and enhanced PGC-1α expression was achieved by the PI3K/Akt and p38 MAPK signaling, we tended to believe that Akt and p38 were “bridges” to link upstream CXXC5 and downstream PGC-1α [39]. In addition, CXXC5 and PGC-1α can further modulate p53 activation, which is responsible for cell-cycle arrest and apoptosis, respectively [40, 41]. However, the expression pattern of CXXC5 and PGC-1α is not always consistent. For instance, CXXC5 negatively regulates Wnt, but lithium, a canonical Wnt/ β-catenin pathway activator, increases PGC-1α expression [42, 43]. Overall, these observations suggested a sophisticated regulatory network involving CXXC5 and PGC-1α.

Collectively, our results revealed that stable CXXC5 overexpression rescued *P. gingivalis*-suppressed cementogenesis by influencing PGC-1α-dependent mitochondrial biogenesis. Mitochondrial biogenesis may be a novel downstream process of CXXC5 in response to *P. gingivalis* infection during cementoblast differentiation.

## Conclusions

The current study identified that CXXC5 is a *P. gingivalis*-sensitive transcription factor that positively regulates cementogenesis by influencing PGC-1α-dependent mitochondrial biogenesis. The in vivo protective role of CXXC5 in cementogenesis during apical inflammation still needs further investigation.

### Supplementary Information

**Additional file 1: Appendix. Appendix Figure 1.** Apical periodontitis mouse models are successfully established. **Appendix Figure 2.** Negative effects of *P. gingivalis* on cementoblast differentiation. **Appendix Figure 3.** Characteristics of lentiviral-transfected OCCM cells. **Appendix Figure 4.** Effects of two transfection methods on downstream gene expression. **Appendix Table 1.** Sources of reagents. **Appendix Table 2.** Primer sequences used for qPCR. Western blots raw images.

## Data Availability

The data used to support the findings of this study are included within the article and the supplementary information file.

## Abbreviations

*P. gingivalis*: *Porphyromonas gingivalis*
CXXC5: CXXC-type zinc finger protein 5
AP: Apical periodontitis
OCN: Osteocalcin
BSP: Bone sialoprotein
ATP: Adenosine triphosphate
PGC-1α: Peroxisome proliferator-activated receptor-γ coactivator 1 alpha
NRF1: Nuclear respiratory factor 1
TFAM: Mitochondrial transcription factor A
qPCR: Quantitative real-time polymerase chain reaction
TSB: Trypticase soy broth
micro-CT: Micro-computed tomography
FBS: Fetal bovine serum
OIM: Osteogenic induction medium
RNAi: RNA interference
NC: Normal control
CCK-8: Cell counting kit-8
ALP: Alkaline phosphatase
ARS: Alizarin red staining
