# Supplementary material for: CXXC5 mitigates P. gingivalis-inhibited cementogenesis by influencing mitochondrial biogenesis
Source: Cell Commun Signal. 2024 Jan 2;22:4. doi: 10.1186/s12964-023-01283-1 (PMC10763120; doi:10.1186/s12964-023-01283-1)
Supplement: Supplementary file 2 — Additional file 1: Appendix. Appendix Figure 1. Apical periodontitis mouse models are successfully established. Appendix Figure 2. Negative effects of P. gingivalis on cementoblast differentiation. Appendix Figure 3. Characteristics of lentiviral-transfected OCCM cells. Appendix Figure 4. Effects of two transfection methods on downstream gene expression. Appendix Table 1. Sources of reagents. Appendix Table 2. Primer sequences used for qPCR. Western blots raw images. [file 12964_2023_1283_MOESM1_ESM.docx]

**CXXC5 Mitigates *P. gingivalis*-inhibited Cementogenesis by influencing Mitochondrial Biogenesis**

Li Ma^1,2^, Huiyi Wang^1^, Xin Huang^1^, Hantao Huang^1^, Yan Peng^1^, Heyu Liu^1^, Xiaoxuan Wang^1,2^,Zhengguo Cao^1,2,*^

^1^State Key Laboratory of Oral & Maxillofacial Reconstruction and Regeneration, Key Laboratory of Oral Biomedicine Ministry of Education, Hubei Key Laboratory of Stomatology, School & Hospital of Stomatology, Wuhan University, Wuhan, China
^2^Department of Periodontology, School & Hospital of Stomatology, Wuhan University, Wuhan, China

**Corresponding Author:**Zhengguo Cao, Department of Periodontology, School & Hospital of Stomatology, Wuhan University, 237 Luoyu Road, Hongshan District, Wuhan, 430079, China.

Tel: +86-27-87686212

Email: [caozhengguo@whu.edu.cn](mailto:caozhengguo@whu.edu.cn)

**Appendix**

**RNA isolation, DNA extraction, and qPCR**

TRIzol ^®^ reagent and PrimeScript RT Reagent Kit were used for isolation of total RNA and reverse transcription, respectively. TaKaRa MiniBEST Universal Genomic DNA Extraction Kit was applied to extract and purify genomic DNA. qPCR was completed on Applied Biosystems QuantStudio 6, with ChamQ SYBR qPCR Master Mix, primers, and templates (complementary-DNA or genomic-DNA). Primers for mouse *Il-6*, *Bsp*, *Osx*, *Ocn*, *Cxxc5*, *Ppargc1a*, *Nrf1*, *Tfam*, *mt-Nd1*, *mt-Cox1*, and *Gapdh* were synthesized by Sangon Biotech (Appendix Table 2). Data were normalized to that of the internal control *Gapdh*, and the 2^-ΔΔCT^ method was used for statistical analysis.

**Western blotting**

Adherent cells were lysed by M-PER mammalian protein extraction reagent, and cellular lysates were centrifugated at 4 °C to remove deposits and to collect total proteins. The loading quantity of each sample was equal (20–40 μg) on SDS-PAGE, and proteins were then transferred onto PVDF membranes (Millipore). After blockade with nonfat milk for 1.5 h, these membranes were incubated with primary antibodies (anti-PGC-1α, anti-NRF1, anti-TFAM, anti-CXXC5, anti-BSP, anti-Osterix, anti-OCN, or anti-β-actin) overnight at 4 °C, and matched secondary antibodies (anti-rabbit and anti-mouse) for 1 h at room temperature. The bands were detected with an enhanced chemiluminescence detection kit or Supersignal West Femto Trial kit and visualized in the Odyssey LI-COR scanner.

**Appendix figures**


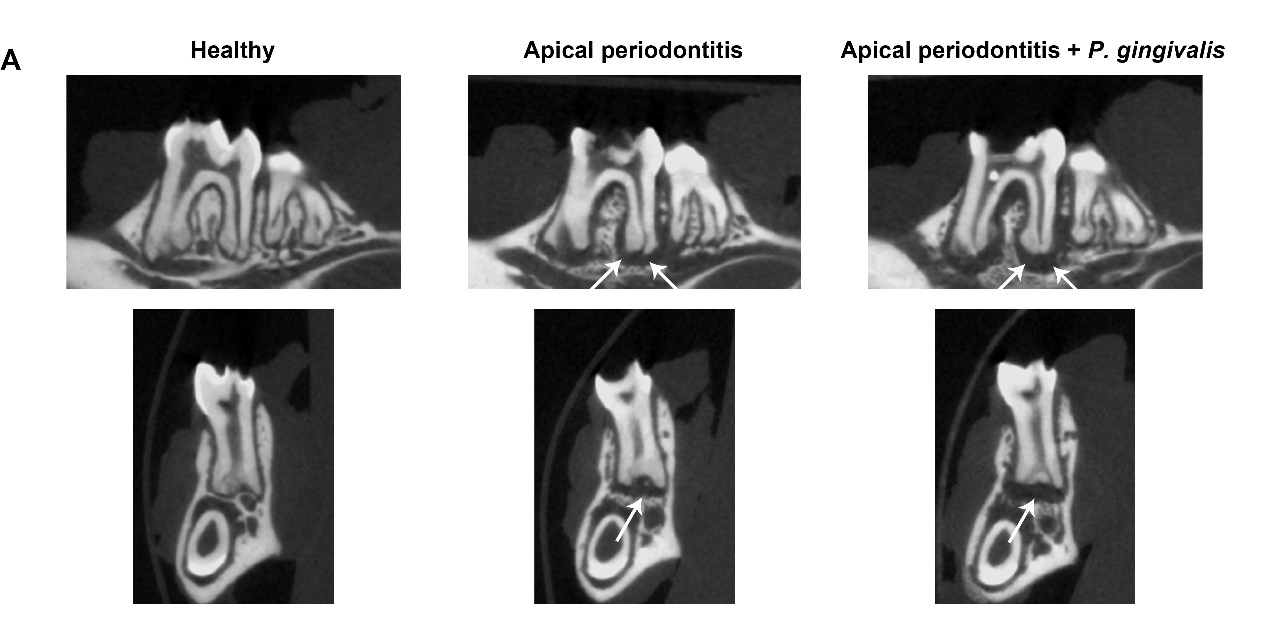
**Appendix Figure 1. Apical periodontitis mouse models are successfully established.**

(A) Micro-CT images showed evident apical bone destruction in the AP group and extended bone resorption in the AP plus *P. gingivalis* group.

**
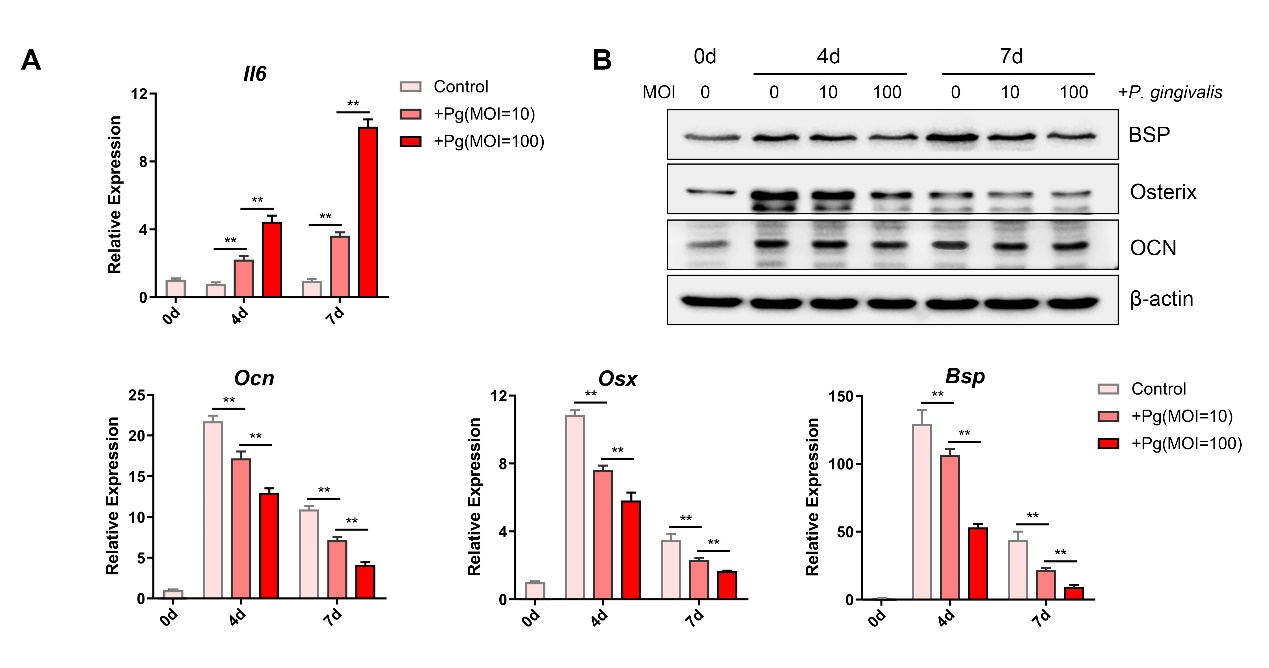
Appendix Figure 2. Negative effects of *P. gingivalis* on cementoblast differentiation.**

(A, B) Cementoblasts were co-cultured with *P. gingivalis* (MOI = 0, 10, 100) for 0, 4, and 7 days. Expressions of *Il-6*, *Ocn*, *Osx*, and *Bsp* was detected by qPCR, and protein levels were displayed by western blotting. Data are the mean ± SD. *P < 0.05, and **P < 0.01.


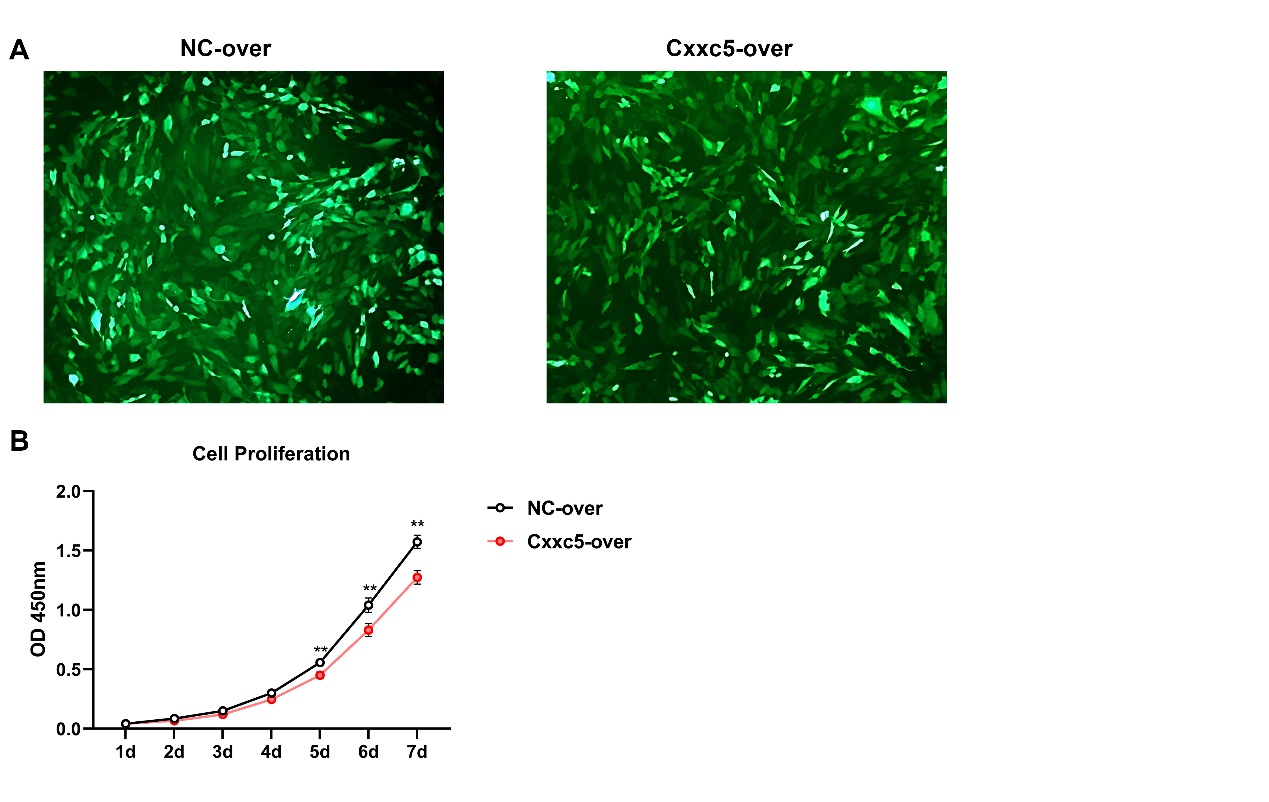
**Appendix Figure 3. Characteristics of lentiviral-transfected OCCM cells.**

(A) Bright green fluorescence was observed to confirm the transfection efficiency after lentiviral transfection of NC-over and Cxxc5-over. (B) Cell proliferation of control and Cxxc5-over cementoblasts was monitored in OIM for 7 days by the CCK-8 assay. Data are the mean ± SD. *P < 0.05, and **P < 0.01.

**
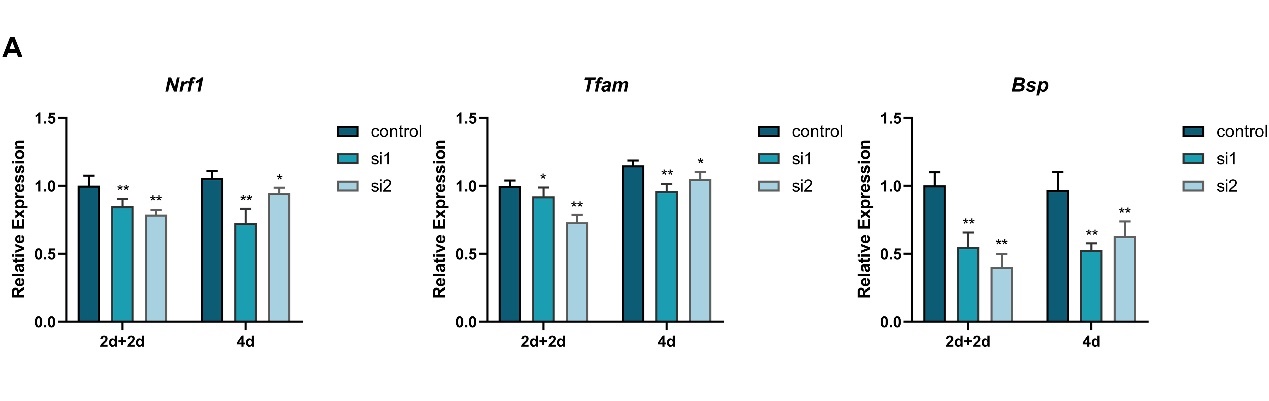
**

**Appendix Figure 4. Effects of two transfection methods on downstream gene expression.**

(A) Effects of two transfection methods and different siRNAs for *Ppargc1a* on mRNA expression of *Nrf1*, *Tfam*, and *Bsp*. Data are the mean ± SD. *P < 0.05, and **P < 0.01.

**Appendix Tables**

**Appendix Table 1. Sources of reagents**

| Reagents | Source | Identifier |
| --- | --- | --- |
| **Chemicals** |  |  |
| tryptic soy broth | BD Bacto, New Jersey, USA | Cat#BD-211825 |
| yeast extract | Oxoid, Basingstoke, UK | Cat#LP0021 |
| menadione | Sangon Biotech, Shanghai, China | Cat#A606528-0005 |
| hemin | Yuanyebio, Shanghai, China | Cat#S19203 |
| blood agar plate | Land Bridge Technology, Beijing, China | Cat#PB003A |
| carboxymethylcellulose sodium | Sigma-Aldrich, St. Louis, MO, USA | Cat#C9481 |
| Hematoxylin staining solution | Servicebio, Wuhan, China | Cat#G1004 |
| Eosin staining solution | Servicebio, Wuhan, China | Cat#G1001 |
| Anti-fade fluorescence mounting medium with DAPI | HelixGen, Guangzhou, China | Cat#HNFD-02 |
| DMEM/High glucose | HyClone, Utah, USA | Cat#SH30243.01 |
| Fetal bovine serum | EVERY GREEN, Hangzhou, China | Cat#13011-8611 |
| Trypsin | HyClone, Utah, USA | Cat#SH30042.01 |
| L-Ascorbic acid | Sigma-Aldrich, St. Louis, MO, USA | Cat#A4544 |
| β-glycerophosphate disodium salt hydrate | Sigma-Aldrich, St. Louis, MO, USA | Cat#G9422 |
| RNAiso Plus (TRIzol) | TaKaRa, Japan | Cat#9108 |
| PrimeScript RT Reagent Kit with gDNA Eraser | TaKaRa, Japan | Cat#RR047 |
| ChamQ SYBR qPCR Master Mix | Vazyme Biotech Co., Ltd., Nanjing, China | Cat#Q311-02 |
| M-PER mammalian protein extraction reagent | Thermo Fisher Scientific, Waltham, MA, USA | Cat#78503-1 |
| Enhanced BCA Protein Assay Kit | Beyotime Biotechnology, Shanghai, China | Cat#P0010 |
| SDS-PAGE kit | Servicebio, Wuhan, China | Cat#G2003-50T |
| Enhanced chemiluminescence detection kit | Advansta Inc., Menlo Park, CA, USA | Cat#K-12045-D10 |
| SuperSignal West Femto trial kit | Thermo Fisher Scientific, Waltham, MA, USA | Cat#34094 |
| BCIP/NBT Alkaline Phosphatase Color Development Kit | Beyotime Biotechnology, Shanghai, China | Cat# C3206 |
| Alizarin Red S | Sinopharm Chemical Reagent, Co., Ltd., Shanghai, China | Cat#71001954 |
| Hexadecylpyridinium chloride monohydrate | Yuanyebio, Shanghai, China | Cat#S48862 |
| Polybrene | HANBIO, Shanghai, China | Cat#HB-PB-1000 |
| INTERFERin transfection reagent | PolyPlus, France | Cat#409 |
| Cell Counting Kit-8 (CCK-8) | Dojindo Molecular Technology, Japan | Cat# CK04 |
| MiniBEST Universal Genomic DNA Extraction Kit Ver.5.0 | TaKaRa, Japan | Cat#9765 |
| CellTiter-Glo Luminescent Cell Viability Assay | Promega, Madison, USA | Cat#G7570 |
| **Antibodies for Western Blotting (Working Concentration)** | |  |
| Anti-PGC-1α (1:1000) | Novus Biologicals, Colorado, USA | Cat#NBP1-04676 |
| Anti-NRF1 (1:1000) | Abcam, Cambridge, England | Cat# ab175932 |
| Anti-TFAM (1:2000) | Proteintech, Wuhan, China | Cat# 22586-1-AP |
| Anti-CXXC5 (1:1000) | Cell Signaling Technology, Danvers, MA, USA | Cat#84546 |
| Anti-BSP (1:1000) | Cell Signaling Technology, Danvers, MA, USA | Cat#5468 |
| Anti-Osterix (1:1000) | Abcam, Cambridge, England | Cat# 22552 |
| Anti-OCN (1:500) | Abcam, Cambridge, England | Cat#ab93876 |
| Anti-β-actin (1:15000) | Proteintech, Wuhan, China | Cat#66009-1-AP |
| Anti-mouse (1:8000) | Proteintech, Wuhan, China | Cat#SA00001-1 |
| Anti-rabbit (1:10000) | Proteintech, Wuhan, China | Cat#SA00001-2 |

**Appendix Table 2. Primer sequences used for qPCR**

| gene | Primer sequences forward/reverse |
| --- | --- |
| *Il-6* | CCACTTCACAAGTCGGAGGCTTA/  CCAGTTTGGTAGCATCCATCATTTC |
| *Bsp* | GAGCCTCGTGGCGACACTTA/  AATTCTGACCCTCGTAGCCTTCATA |
| *Osx* | CCTCTCGACCCGACTGCAGATC/ AGCTGCAAGCTCTCTGTAACCATGAC |
| *Ocn* | GAGGACCATCTTTCTGCTCACT/  CGGAGTCTGTTCACTACCTTATTG |
| *Cxxc5* | AGGCAGGAGGAACAGACA/  GTGCTTTGAGGTAGGGTTG |
| *Ppargc1a* | GAATCAAGCCACTACAGACACCG/ CATCCCTCTTGAGCCTTTCGTG |
| *Nrf1* | GGCAACAGTAGCCACATTGGCT/  GTCTGGATGGTCATTTCACCGC |
| *Tfam* | ATTCCGAAGTGTTTTTCCAGCA/  TCTGAAAGTTTTGCATCTGGGT |
| *mt-Nd1* | TCAACCCTAGCAGAAACAAACC/  GGCCGGCTGCGTATTCTAC |
| *mt-Cox1* | TCAGTATCGTATGCTTCAACAAATTTAGA/  TGGTTCCTCGAATGTGTGATATG |
| *Gapdh* | GGAGATTGTTGCCATCAACGA/ GAAGACACCAGTAGACTCCACGACA |

**
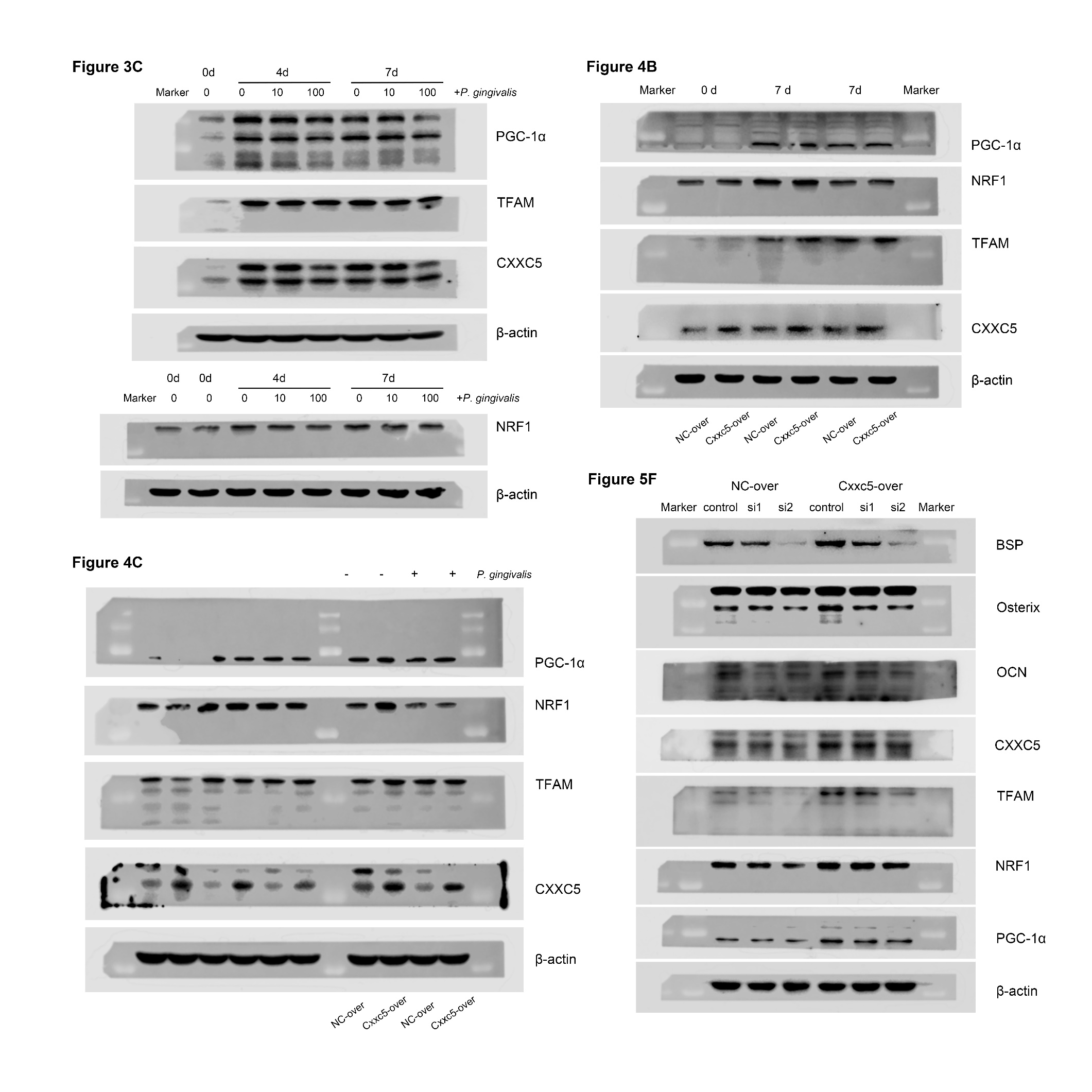

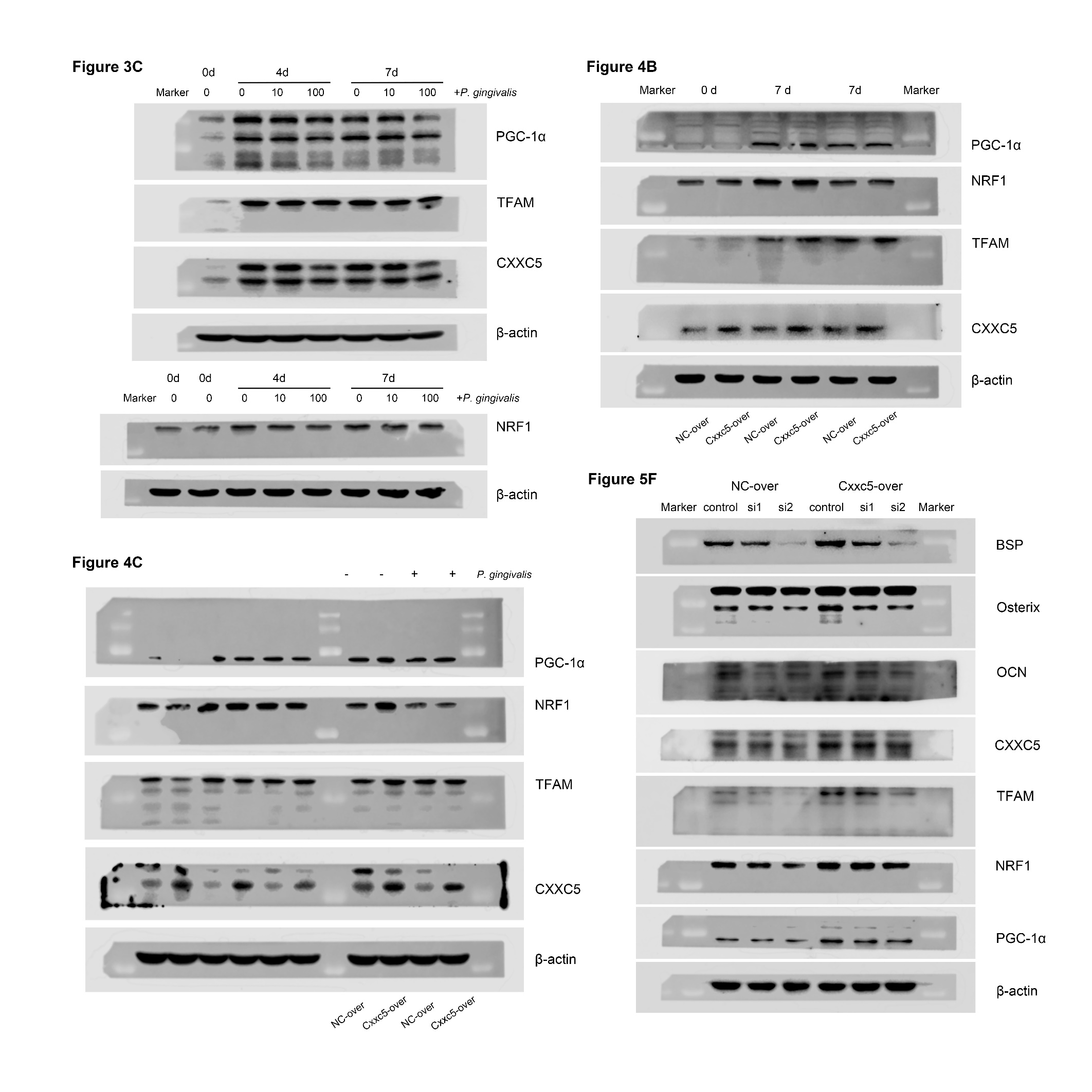
Western blots raw images**

**
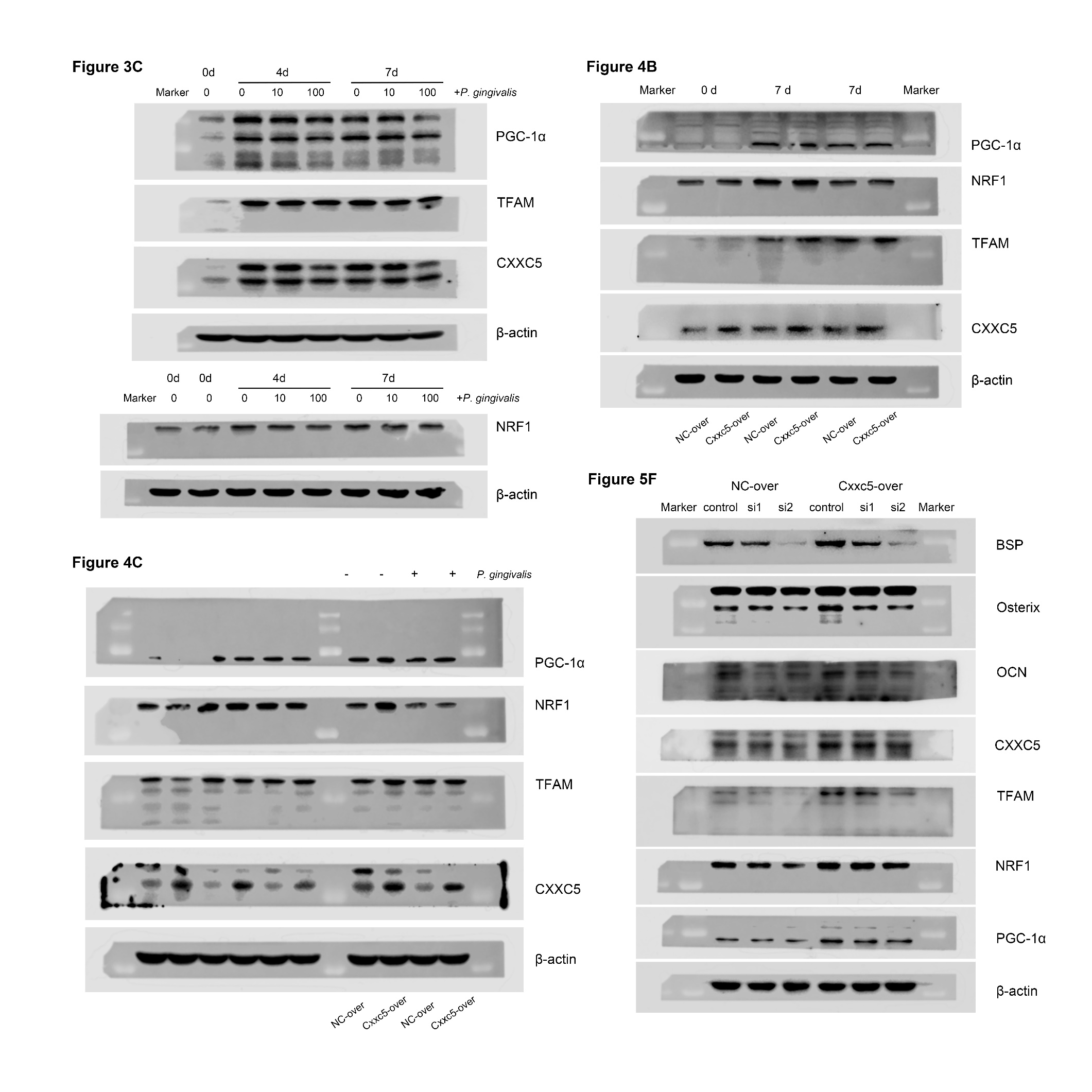

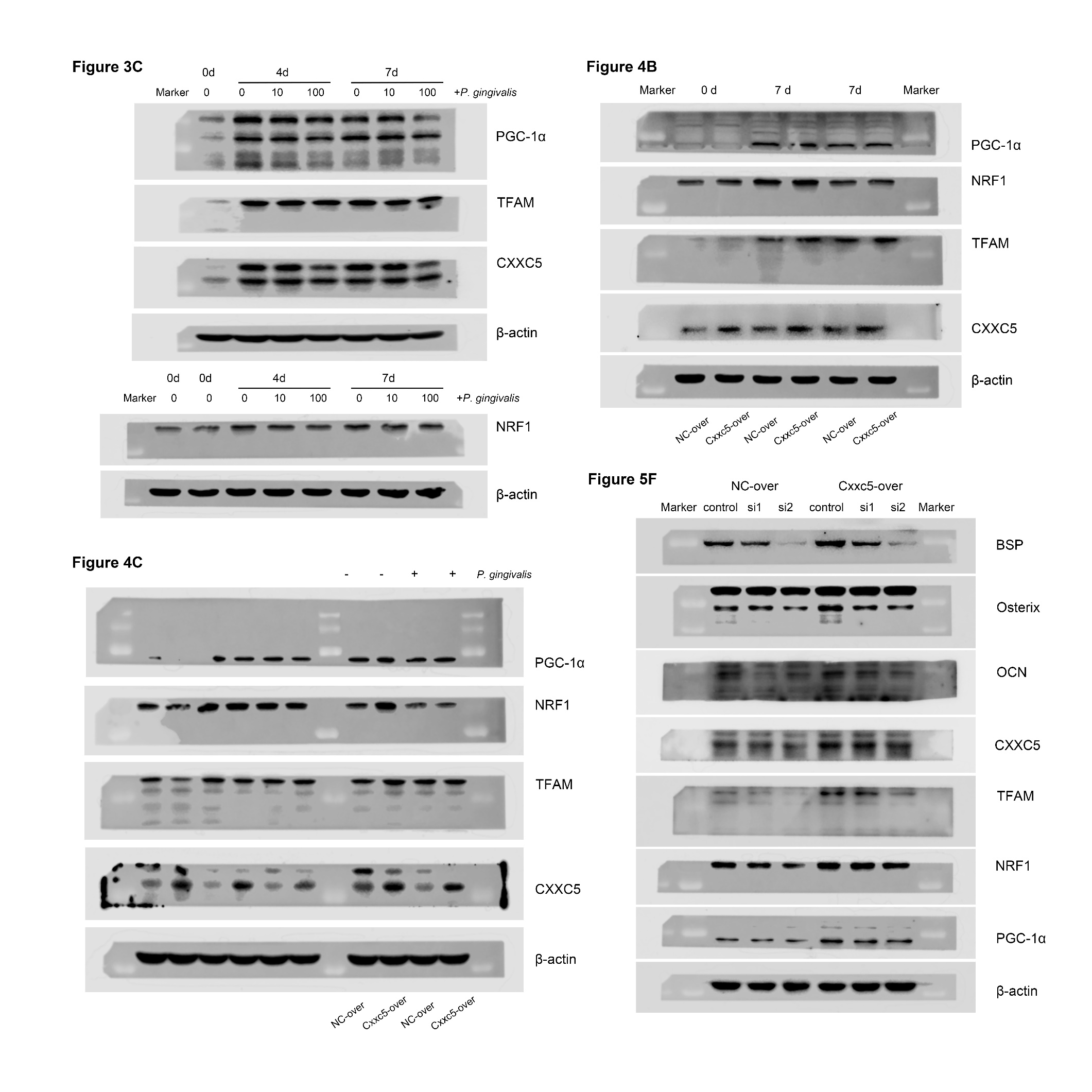
**
